# Interconnecting EDOT-Based Polymers with Native Lignin toward Enhanced Charge Storage in Conductive Wood

**DOI:** 10.1021/acsami.4c16298

**Published:** 2024-12-03

**Authors:** Van Chinh Tran, Gabriella Mastantuoni, Jonas Garemark, Christopher H. Dreimol, Xin Wang, Magnus Berggren, Qi Zhou, Renee Kroon, Isak Engquist

**Affiliations:** † Laboratory of Organic Electronics, Department of Science and Technology, Linköping University, SE-601 74, Norrköping, Sweden; ‡ Wallenberg Wood Science Center, Department of Science and Technology, Linköping University, SE-601 74 Norrköping, Sweden; § Wallenberg Initiative Material Science for Sustainability, Department of Science and Technology, Linköping University, SE-601 74 Norrköping, Sweden; ∥ Department of Chemistry, Massachusetts Institute of Technology, 77 Massachusetts Avenue, Cambridge, Massachusetts 02139, United States; ⊥ Division of Glycoscience, Department of Chemistry, KTH Royal Institute of Technology, AlbaNova University Centre, 106 91 Stockholm, Sweden; # Wallenberg Wood Science Center, Department of Fiber and Polymer Technology, KTH Royal Institute of Technology, 100 44 Stockholm, Sweden; ¶ Wood Materials Science, Institute for Building Materials, ETH Zürich, 8093 Zürich, Switzerland; ∇ Cellulose & Wood Materials Laboratory, Empa, 8600 Dübendorf, Switzerland; ○ Division Digital Systems, Department Smart Hardware, Unit Bio- and Organic Electronics, RISE Research Institutes of Sweden, 602 33, Norrköping, Sweden; ⧫ Digital Cellulose Center, RISE, 602 33 Norrköping, Sweden

**Keywords:** wood, organic electronics, PEDOT, lignin, energy storage

## Abstract

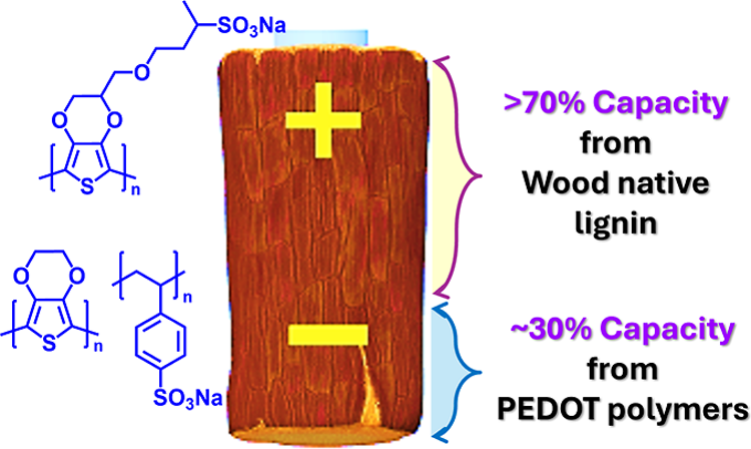

The 3D micro- and nanostructure of wood has extensively been employed as a template for cost-effective and renewable electronic technologies. However, other electroactive components, in particular native lignin, have been overlooked due to the absence of an approach that allows access of the lignin through the cell wall. In this study, we introduce an approach that focuses on establishing conjugated-polymer-based electrical connections at various length scales within the wood structure, aiming to leverage the charge storage capacity of native lignin in wood-based energy storage electrodes. We demonstrate that poly(3,4-ethylenedioxythiophene)–poly(styrenesulfonate) PEDOT/PSS, integrated within the cell wall lumen, can be interfaced with native lignin through the wood cell wall through in situ polymerization of a water-soluble S-EDOT monomer. This approach increases the capacitance of the conductive wood to 315 mF cm^–2^ at a scan rate of 5 mV s^–1^, which is seven and, respectively, two times higher compared to the capacitance of conductive wood made with the single components PEDOT/PSS or S-PEDOT. Moreover, we show that the capacitance is contributed by both the electroactive polymers and native lignin, with native lignin accounting for over 70% of the total charge storage capacity. We show that accessing native lignin through in situ creation of electrical interconnections within the wood structure offers a pathway toward sustainable, wood-based electrodes with improved charge-storage capacity for applications in electronics and energy storage.

## 1 Introduction

Wood has been a fundamental structural material throughout human history and still retains its pivotal position in today’s industrial areas, including paper manufacturing, furniture production, and construction. Thanks to the advancements in science and technology, micro- and nanostructured wood has been developed with novel functionalities for high-tech applications^1,2^ in photonic^3,4^ and electronic^2,5^ devices. With regards to electronic applications, wood has been carbonized or modified with electroactive materials to form conducting wood electrodes^5−8^ in supercapacitors,^9^ batteries,^10^ and transistors.^11^ Unfortunately, carbonization disregards the advantage of redox-active wood components (i.e., lignin) and raises environmental concerns due to the energy-intensive process that requires temperatures of several hundred to 1000 °C.^5,6^ These issues can be overcome by utilizing wood in its native form, which offers redox-active activities from native lignin^12^ and facilitates ionic transport at different length scales.^11,13^ Consequently, noncarbonized wood has recently emerged as a promising template for the preparation of conductive wood-based electronics and energy storage devices.^4,12,14^

While lignin has been recognized as a potential redox-active material in supercapacitors^12,15^ and batteries^16^ most reported conductive wood electrodes have overlooked its role in charge storage capacity. This oversight persists due to the absence of an approach that efficiently utilizes native lignin and demonstrates its contribution to the total capacitance of conductive wood. Recent studies have shown promising redox activities of biorefined lignin when combined with graphene,^17^ carbon derivatives,^16,18^ and conducting polymers^15,19^ in energy storage devices. Consequently, the inclusion of these conductive materials in wood is expected to access and utilize the redox properties of native lignin within the wood structure. This was shown in our previous work,^12^ where we incorporated poly(3,4-ethylenedioxythiophene)–poly(styrenesulfonate) (PEDOT/PSS) into sulfonated wood, aiming to utilize the charge storage capacity of lignin in the total capacitance of a wood-based supercapacitor.

However, it is essential to create stable interconnecting electrical pathways throughout the wood cell wall when aiming to efficiently access and utilize native lignin distributed across different length scales of wood tissue. For example, PEDOT/PSS has limitations due to its large particle size,^12,20^ which prevents the formation of electronic pathways through the cell wall and limits the utilization of lignin inside the cell wall and central middle lamella.^12^ As a result, conductive wood prepared with PEDOT/PSS exhibits limited charge storage capacitance (38 mF cm^–2^).^12^ In a previous study,^21^ we in situ polymerized polypyrrole (PPy) in sulfonated wood, successfully introducing it to the wood cell wall. The obtained electrode exhibited an improved capacitance but suffered a large capacitance reduction due to the poor electrochemical stability of PPy. Furthermore, the effectiveness of PPy in utilizing native lignin remained inconclusive, as there was insufficient electrochemical evidence to confirm the successful utilization of native lignin’s capacity.^21^ To address these challenges, an ideal approach should be developed that encompasses highly conductive, electrochemically stable materials like PEDOT polymers^22^ that can be in situ polymerized across different length scales of wood tissues, from microscopic cell wall channels to nanoscale wood fibers for effective utilization of lignin.

In this work, we have developed such an approach and synergistically use two PEDOT polymers, PEDOT/PSS and S-PEDOT,^23^ in conductive wood. With this approach, we demonstrate that redox-active lignin can be interconnected with electrically conducting pathways of PEDOT/PSS in the lumen via the in situ-polymerized S-PEDOT monomer (S-EDOT) within the wood cell wall and the central middle lamella. We show that the water-soluble S-EDOT monomer allows for easy distribution throughout the wood and that the S-PEDOT polymer is located in the cell wall and central middle lamella. We find that the synergistic use of these two polymers significantly enhances the electrode capacitance, achieving capacitances that are more than 2 and 7 times higher than those of conductive woods using only one polymer, S-PEDOT/wood or PEDOT/PSS/wood, respectively. This improvement can be attributed to the interconnected network that is created between the three electroactive materials, native lignin, S-PEDOT, and PEDOT/PSS throughout the conductive wood structure. The interconnectivity and distribution of these materials in wood are verified by SEM and confocal Raman spectroscopy. The obtained conductive wood electrode has shown a capacitance of 315 mF cm^–2^ at the scan rate of 5 mV s^–1^ with contribution from both the polymers and native lignin. Notably, native lignin accounts for over 70% of the capacitance of the overall charge storage capacity of conductive wood. Our results highlight the effectiveness of our approach in utilizing native lignin’s capacity using a small fraction (∼6 wt %) of polymers in wood. We expect our work to demonstrate the efficacy of a multimaterial approach for the next generation of sustainable wood-based electronics and energy storage devices.

## 2 Results and Discussion

### 2.1 Approach for Interconnecting Lignin with Electroactive Components

**Figure 1:**
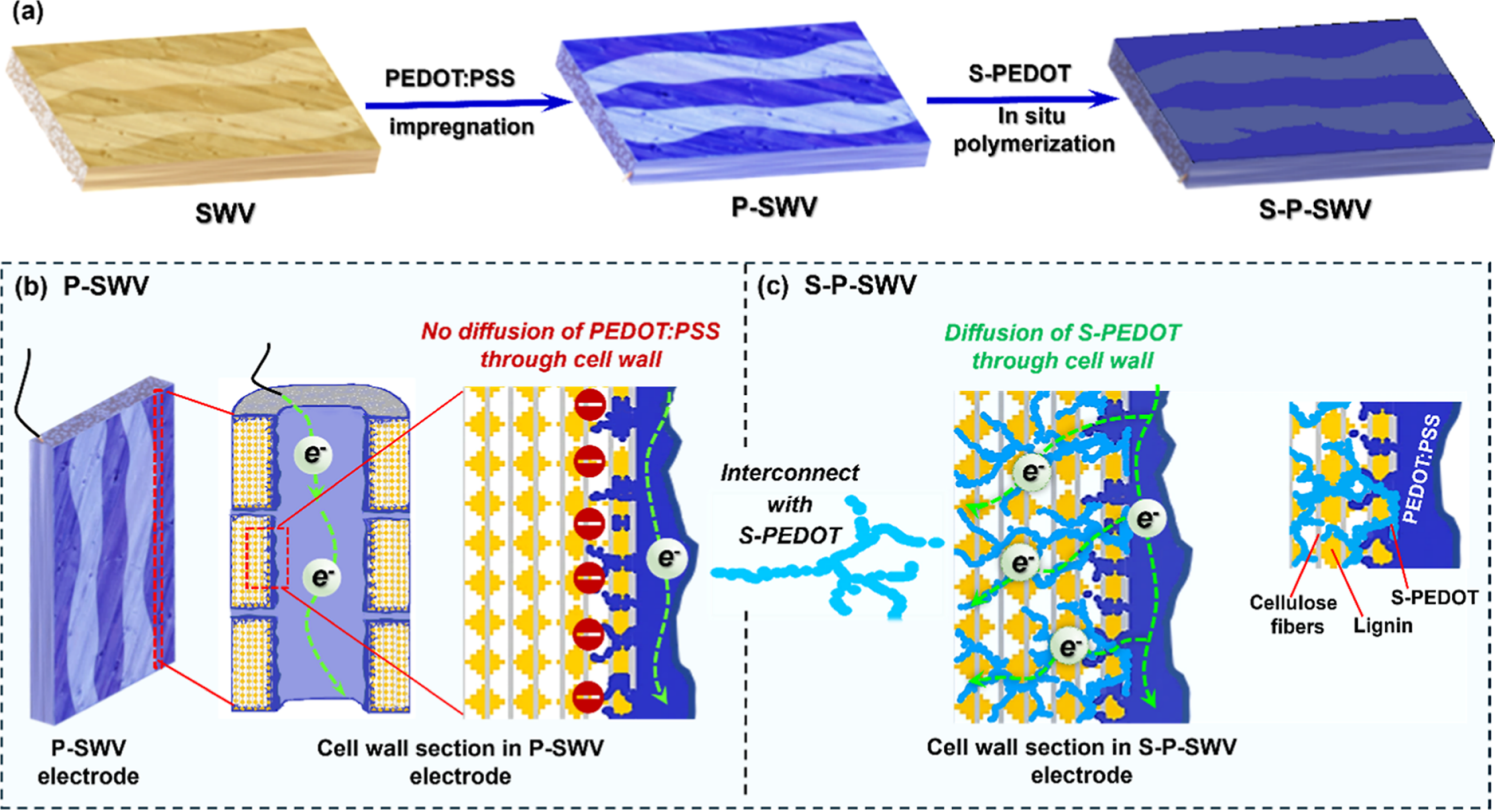
(a) Schematic of the conductive wood preparation process, (b) cell wall section in P-SWV electrode, showing no electrical pathways through the cell wall for lignin redox chemistry, and (c) cell wall section in S-P-SWV electrode, showing S-PEDOT-based electrical pathways through the cell wall for lignin redox chemistry.

The synthetic route of conductive wood consists of three main steps: wood sulfonation, impregnation, and in situ polymerization (Figures 1a, and S2a, Supporting Information). Wood sulfonation is required to open the wood structure and facilitates the ingression of the electroactive components while preserving the native lignin content at about 17 wt %.^12,21^ The electroactive polymers, PEDOT/PSS and S-PEDOT, were subsequently introduced into the obtained sulfonated wood veneer (SWV), where each polymer fulfills a specific function. PEDOT/PSS was primarily utilized to create electrical conduction at the micrometer scale of the cell wall lumen, whereas S-PEDOT was mainly used to electrically connect PEDOT/PSS to the submicrometer- or nanostructure inside the wood cell wall and central middle lamella (Figure 1b,c). Although PEDOT/PSS was used for wood-based energy storage devices in a previous report, its grain-shell structure, with a diameter of about 300–500 nm,^20^ limited its ability to access lignin inside the wood cell wall (Figure 1b), where pore sizes typically range from 3 to 50 nm.^21^ Consequently, the lignin inside the wood cell wall and central middle lamella was not sufficiently utilized, as there is no charge transport in this lignin-rich region (Figure 1b).

To improve the interconnectivity of lignin with PEDOT/PSS, we employ in situ polymerization of S-PEDOT,^23^ which allows the water-soluble S-EDOT monomer to first penetrate throughout the wood structure prior to its polymerization. The connection of the PEDOT/PSS thin film layer in the cell wall lumen (considered the main pathway for electronic charges) with the S-PEDOT polymer networks grown inside the wood cell wall (considered to be the interconnecting pathways between lignin and PEDOT/PSS) is anticipated to facilitate electronic charge transport throughout the entire wood structure (Figure 1c). The complete electroactive structure should facilitate lignin redox activity and consequently utilize its charge storage capacity. While this approach is expected to be applicable to various wood types, pine veneer was chosen for this study. In previous work,^11,12^ we prepared conductive woods by impregnating different wood veneers (WVs), including balsa, pine, birch, and ash, with PEDOT/PSS. Among these, the pine-based conductive wood demonstrated the highest conductivity.^12^ Moreover, pine is a softwood with a high lignin content, making it particularly well-suited for focusing on native lignin utilization in this work.

### 2.2 Structure and Morphology of Sulfonated and Conductive WVs

**Figure 2:**
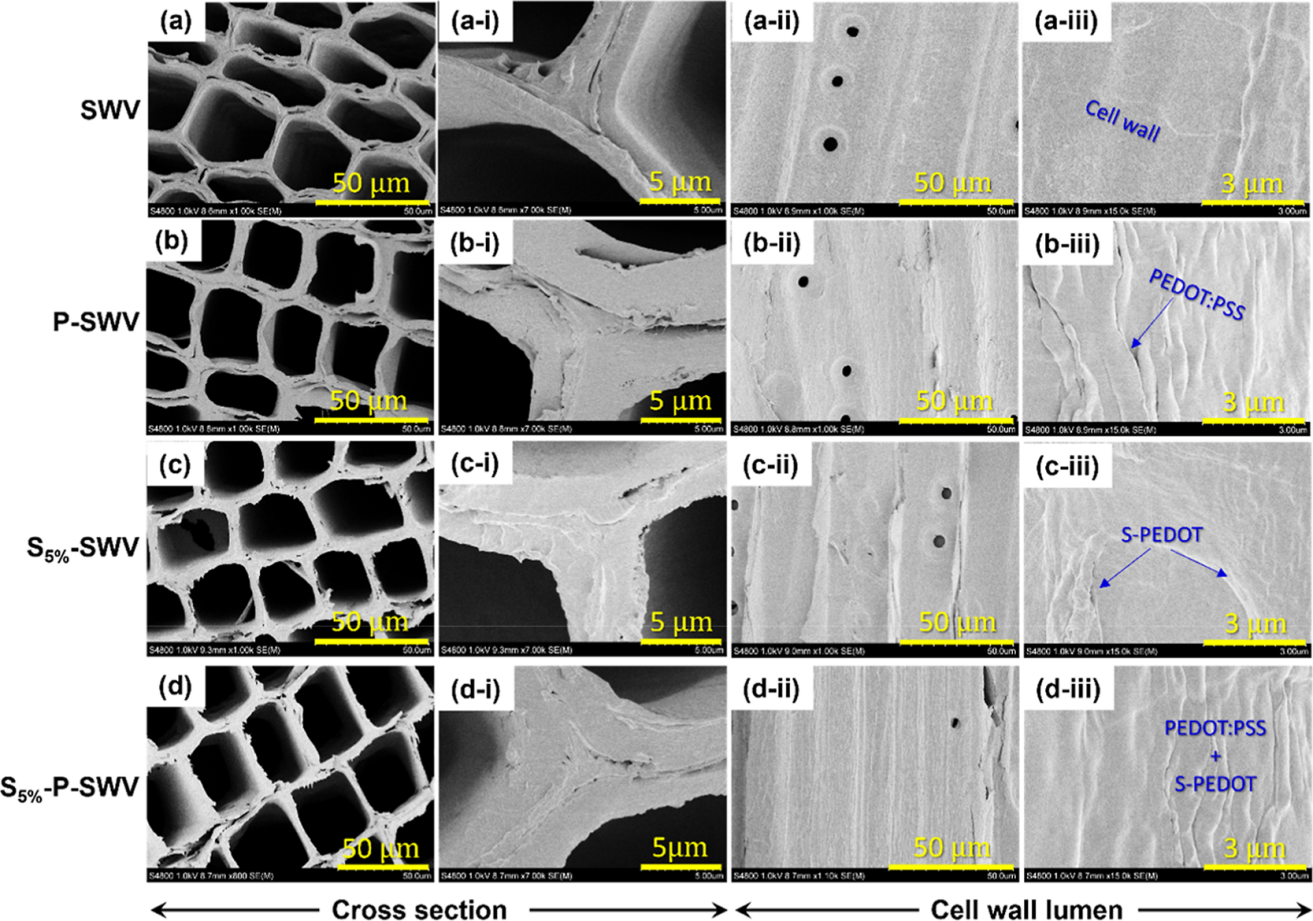
SEM images showing the morphological structure of wood samples. The cross-section of SWV (a,a(i)), P-SWV (b,b(i)), S_5%_-SWV (c,c(i)), and S_5%_-P-SWV (d,d(i)). The inner surface of the cell wall lumen of SWV (a(ii),a(iii)), P-SWV (b(ii),b(iii)), S_5%_-SWV (c(ii),c(iii)), and S_5%_-P-SWV (d(ii),d(iii)).

The preparation of SWV was reported in previous work,^12^ where SWV presented a higher porosity than native wood, and lignin was preserved inside the wood structure at a content of about 17 wt %.^12^ The high porosity and lignin content make SWV a suitable porous template for incorporating PEDOT/PSS or S-PEDOT through impregnation or in situ polymerization processes, respectively. In this study, the morphological structure of SWV and conductive WVs was characterized using SEM (Figure 2), showing the cross sections (Figure 2a–d,a(i)–d(i)) and the inner surface of the cell wall lumen (Figure 2a(ii)–d(ii),a(iii)–d(iii)). In Figure 2a,a(i), the cross-section of the SWV cell wall presents an intact structure with sparse opening sections occurring at the border, the corner, or the central middle lamella between the wood cell walls. In addition, the cell wall lumen of SWV (Figure 2a(ii,iii)) presented a clear and smooth inner surface similar to the native wood.^12,21^

Next, we impregnated SWV with PEDOT/PSS, resulting in the conductive wood P-SWV. Upon its impregnation with PEDOT/PSS, P-SWV presented a similar cell wall structure as seen in SWV (Figure 2b,b(i)). However, the inner surface of P-SWV’s cell wall lumen presented distinct wrinkled layers of PEDOT/PSS, indicating the successful deposition of a PEDOT/PSS layer on the lumen surface (Figure 2b(ii,iii)). This observation is also supported by the color change from yellow in the SWV sample to blue in the P-SWV sample (Figure S2a in the Supporting Information).

We also performed in situ polymerization of S-EDOT (5 wt % solution) in SWV, after which S_5%_-SWV was obtained. We mainly focused on polymerizing S-EDOT in a 5 wt % solution, as it was proven to be an optimal condition for preparing the high-conductivity S-PEDOT polymer.^23^ After polymerization, we observed a similar color change from yellow to dark blue (Figure S2b, Supporting Information), indicating successful polymerization of S-EDOT in SWV. The superior diffusion of S-PEDOT into the wood structure, compared to PEDOT/PSS, is visually evident when comparing the color homogeneity of S_5%_-SWV and P-SWV (see Figure S2). In Figure 2c(ii,iii), S-PEDOT layers were found on the lumen inner surface, forming a slightly smoother layer compared to the PEDOT/PSS layers. We attributed this observation to the smaller particle size of S-PEDOT compared to PEDOT/PSS.^20,23^ While S_5%_-SWV presented a similar cell wall structure as in SWV and P-SWV, sparse thin films were found partly blocking the hollow space of S_5%_-SWV’s cell wall lumens (Figure 2c). These films are probably S-PEDOT polymer films formed during the in situ polymerization process.

We now turn to samples containing both PEDOT/PSS and S-PEDOT. Due to PEDOT/PSS having notably larger particle sizes than S-PEDOT,^20,23^ the composite formed by these two polymers should display a morphological structure akin to that of PEDOT/PSS. This is indeed the case, as the SWV was initially coated with PEDOT/PSS before undergoing in situ polymerization to incorporate S-PEDOT. Consequently, the cell wall inner surface of the resulting conductive wood, S_5%_-P-SWV, exhibits a polymer layer with a morphological structure highly similar to that of PEDOT/PSS (see Figure 2d(ii,iii)).^12^ The S_5%_-P-SWV’s cell wall structure is near-identical, as observed for S_5%_-SWV and P-SWV. Consequently, the successful combination of these two polymers is only evidenced by visualizing the change of yellow color SWV to blue in P-SWV and a dark blue color in the S_5%_-P-SWV sample (see Figure S2a, in the Supporting Information).

**Figure 3:**
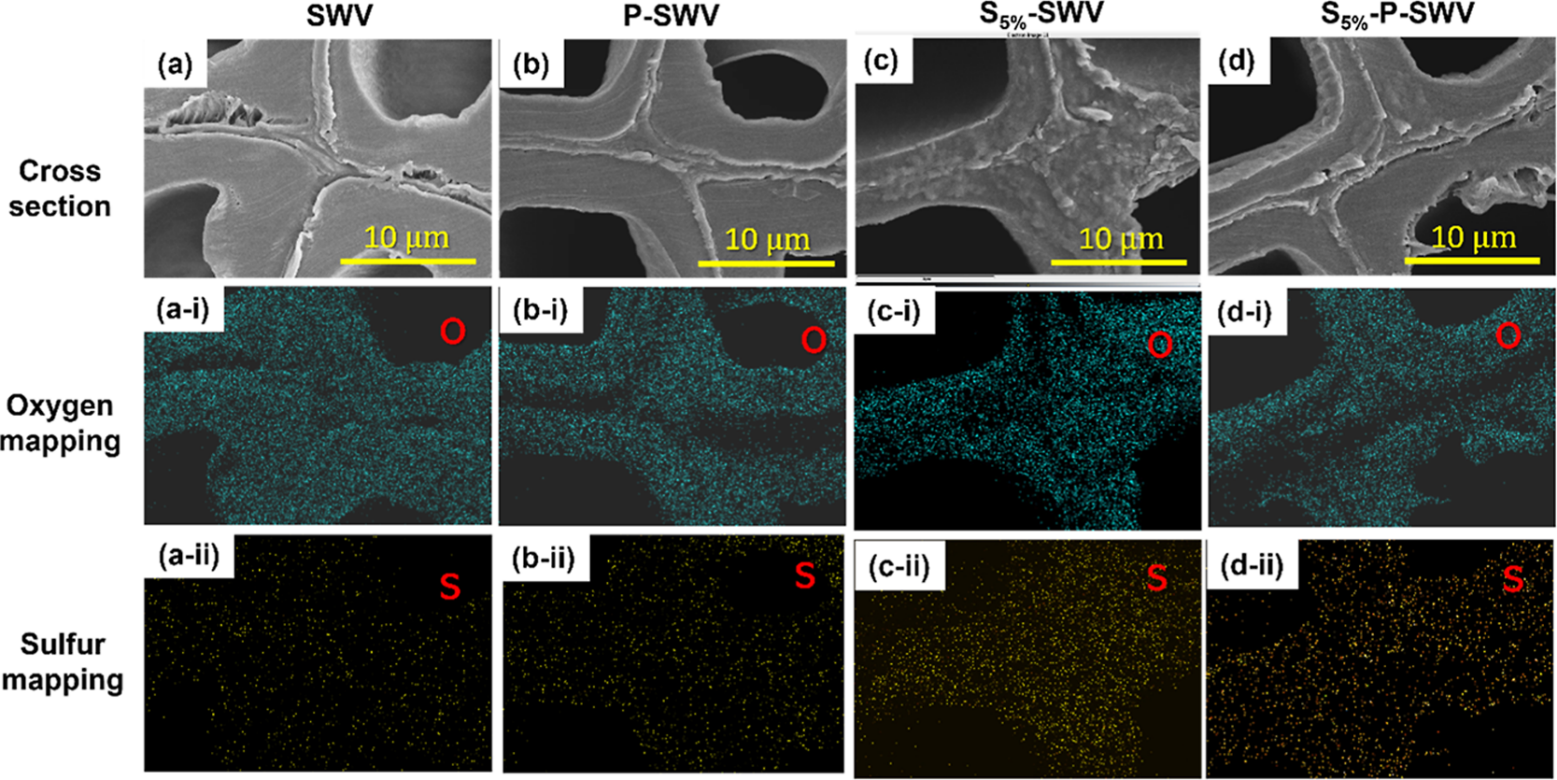
SEM and EDX-elemental mapping images of wood samples: the SEM images show the cross-section of SWV (a), P-SWV (b), S_5%_-SWV (c), and S_5%_-P-SWV (d). Elemental mapping images of oxygen in the cross-section of SWV (a(i)), P-SWV (b(i)), S_5%_-SWV (c(i)), and S_5%_-P-SWV (d(i)). Elemental mapping images of sulfur in the cross-section of SWV (a(ii)), P-SWV (b(ii)), S_5%_-SWV (c(ii)), and S_5%_-P-SWV (d(ii)).

Although both polymers could be identified on the surface of the wood cell wall lumen, S-PEDOT is anticipated to penetrate the entire wood structure. To examine the distribution of S-PEDOT inside the cell wall and central middle lamella, EDX elemental mapping was employed to explore the distribution of sulfur in SWV and its corresponding conductive woods.^11^ The obtained SEM (Figure 3a–d) and EDX mapping of oxygen (Figure 3a(i)–d(i)) and sulfur (Figure 3a(ii)–d(ii)) show that SWV can be seen to contain only a small amount of sulfur (Figure 3a(ii)). The existing sulfur is due to the wood sulfonation process. As SWV is combined with PEDOT/PSS, S-PEDOT, or both of these polymers, the intensity of the sulfur peak is increased in P-SWV (Figure 3b(ii)) and reaches the highest intensities in S_5%_-SWV (Figure 3c(ii)) and S_5%_-P-SWV (Figure 3d(ii)). EDX compositional analysis (Figure S4, Supporting Information) showed 0.8, 3.3, 5.0, and 5.4 at. % of sulfur in the SWV, P-SWV, S_5%_-SWV, and S_5%_-P-SWV samples, respectively. These average values were measured for the whole mapping area (Figure 3), ensuring a fair comparison of sulfur content between these samples.^11,21^ Since the mapping areas focused on the cell wall and central middle lamella, the higher sulfur composition in S_5%_-P-SWV (5.4 at. %) compared to P-SWV (3.3 at. %) is interpreted as an indication of the presence of S-PEDOT in the wood cell wall and the central middle lamella of S_5%_-P-SWV. The atomic composition of sulfur (S) in S_5%_-P-SWV is only slightly higher (5.4 at %) than in S_5%_-SWV (5.0 at. %). This suggests that only a limited quantity of S-PEDOT can be incorporated into PEDOT:PSS-containing conductive wood (P-SWV).

**Figure 4:**
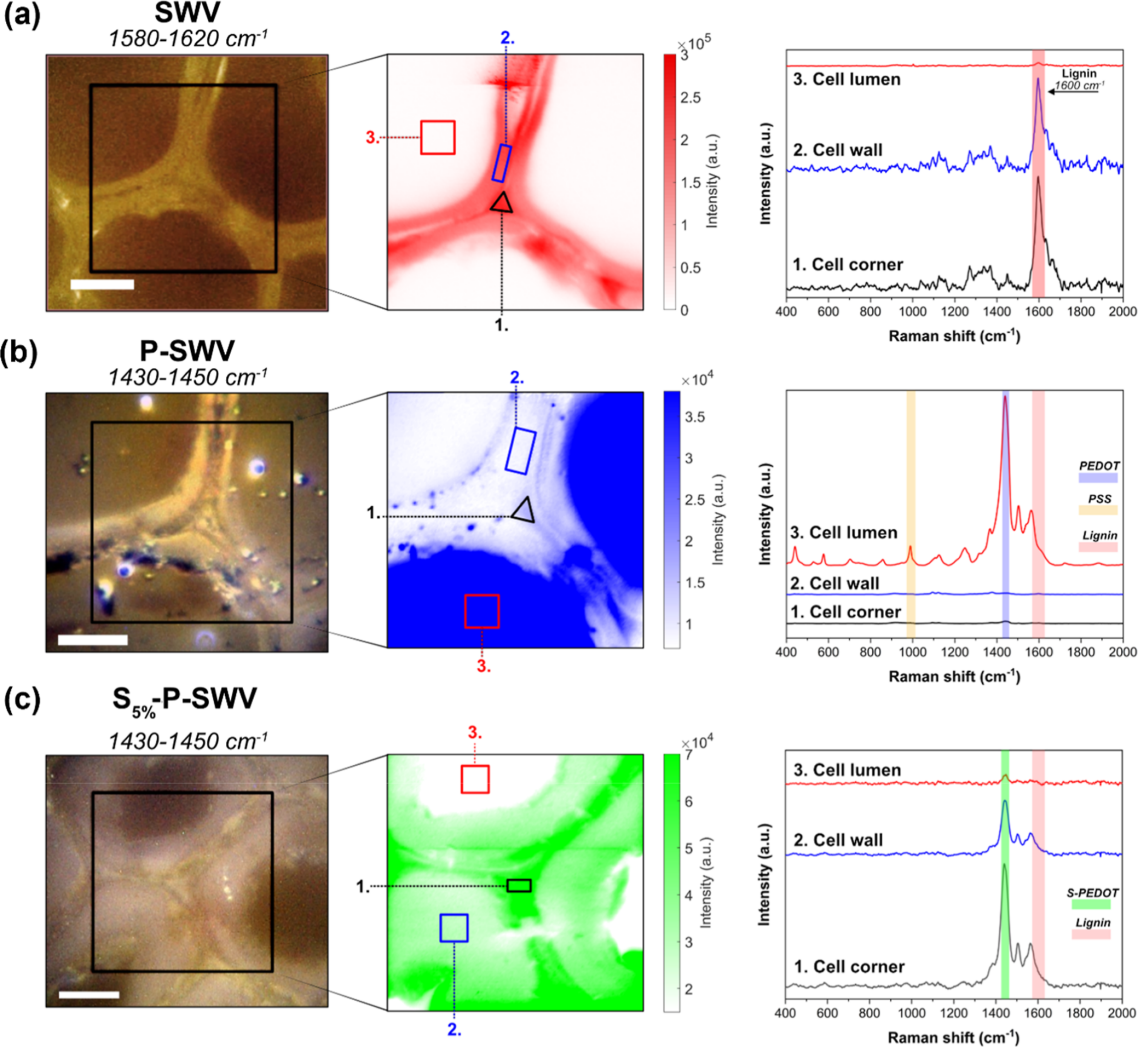
Confocal Raman microscopy of wood samples: (a) microscope image of sulfonated wood (SWV) accompanied by a Raman map showing the lignin signal in red, followed by signals from the individual regions of interest which were integrated from the areas of the map. In (b,c), maps show the EDOT signal between 1430 and 1450 cm^–1^ of the conductive woods (b) (P-SWV) and (c) S_5%_-P-SWV.

To confirm the presence of S-PEDOT in the wood cell wall and the central middle lamella, confocal Raman microscopy was employed, aiming to distinctively map the distribution of PEDOT/PSS and S-PEDOT in the S_5%_-P-SWV conductive wood structure (Figure 4).^7,24^ We observe that the bare sulfonated wood (SWV) (Figure 4a) shows a substantial presence of lignin within the cell corner (1) and cell wall (2) but that the lumen that is absent of any lignin signal, highlighted through the color map shown in the middle panel, where red indicates the presence of lignin.^7,24^ The following Raman map in Figure 4b, of the PEDOT/PSS-containing conductive wood (P-SWV), shows a complete filling of the lumen structure with PEDOT/PSS (blue color in the middle panel), with some additional presence through the cell pits and outer cell wall.^7^ However, PEDOT/PSS infiltration is very minor in the secondary cell wall and cell corner, as seen in the Raman spectra.^7^ It should be noted that the amount of lignin is with high probability the same as for the sample in a); however, the lignin-related Raman peaks are substantially weaker compared to the PEDOT-related peaks and therefore cannot be distinguished in the spectra shown in the rightmost b) panel. In Figure 4c, we clearly see that the S-EDOT infiltration and polymerization to S-PEDOT results in homogeneous distributions within the cell wall, cell corner, and middle lamella of S_5%_-P-SWV, respectively. The small PEDOT peak in region 3 (cell lumen) mainly corresponds to the PEDOT/PSS that is filling the lumen. Scaling to accommodate the very strong signals from the S-PEDOT makes this peak appear smaller than in (b); however, there is reason to believe that all the PEDOT/PSS is remaining in the lumen, partly because of the SEM–EDX results presented above and partly because the accompanying PSS peaks can still be seen if the spectrum is magnified.^7^ Remarkably, the S-PEDOT signal is strong throughout the large latewood cell wall of Figure 4c, which indicates a complete filling of all cell walls throughout the WV. Supporting Information Figure S5 shows the mapping of an earlywood structure of S_5%_-P-SWV. Taken together, the confocal Raman measurements strongly support the conclusion that PEDOT/PSS only infiltrates the cell lumen, whereas S-PEDOT is able to penetrate the cell wall, thereby creating a synergistic effect where as much as possible of the lignin is contacted electrically while also maintaining good electrical conductivity on a macroscopic scale.

### 2.3 Electrical and Electrochemical Properties of CWV Wood Electrodes

The electrical conductivity of different conductive woods was measured using a four-point probe technique, as reported in previous studies.^7,11,21^ For the S_5%_-SWV conductive wood, we measure electrical conductivity of 59 ± 5 S/m, which is lower than that of P-SWV (104 ± 10 S/m) and S_5%_-P-SWV (141 ± 12 S/m). The lower conductivity in S_5%_-SWV is attributed to the inferior electrical conductivity of S-PEDOT (80 ± 5 and 192 ± 8 S/cm for films prepared from 1 and 5 wt % S-PEDOT suspensions, respectively) compared to PEDOT/PSS (∼850 S/cm^12^). However, the synergistic combination of these two polymers in wood has resulted in the highest conductivity observed for the S_5%_-P-SWV conductive wood. This result suggests that our approach holds great potential for the preparation of highly conductive wood for electronics applications.^11,14,25^

**Figure 5:**
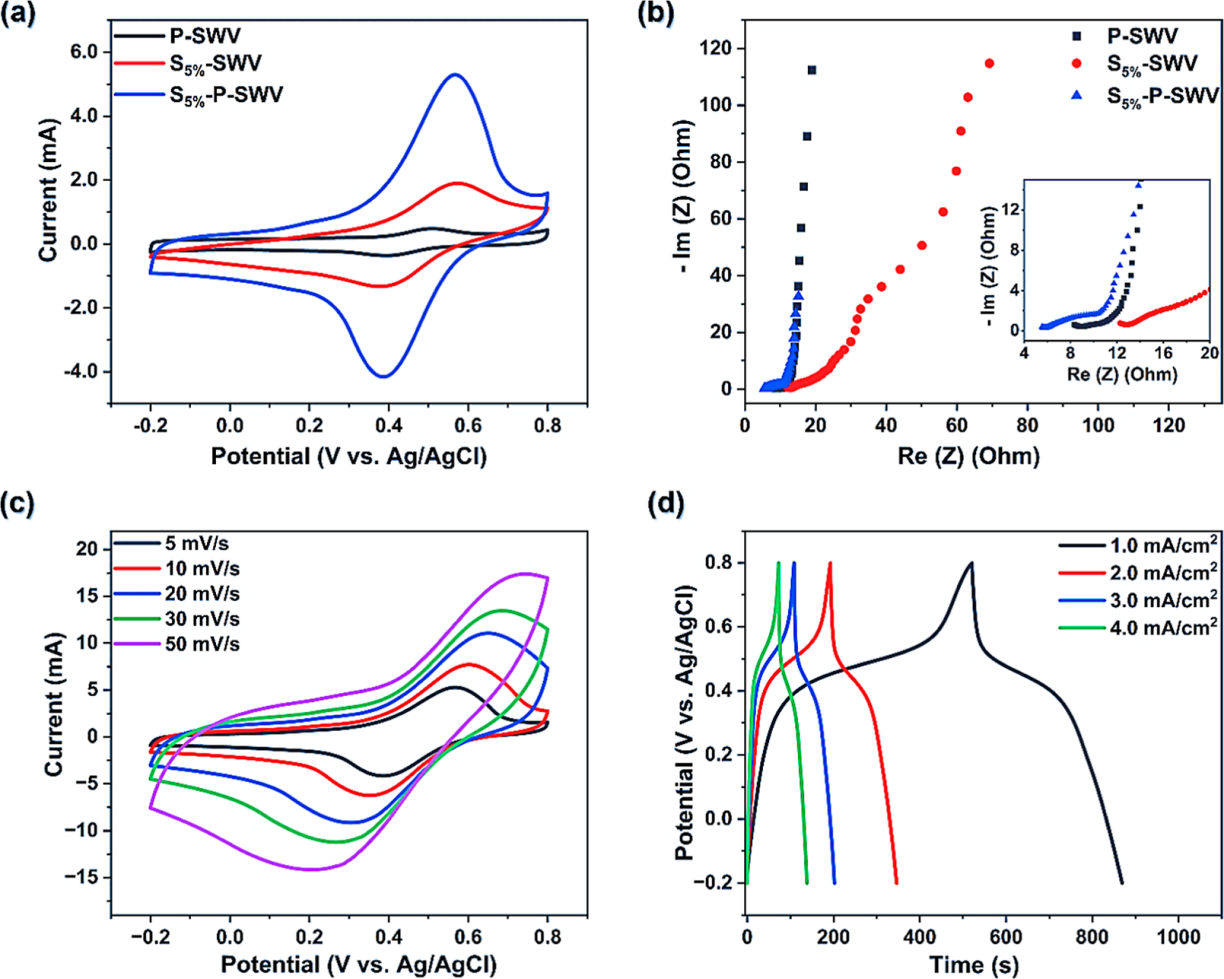
Electrochemical properties of conductive wood electrodes: (a) CV curves at 5 mV s^–1^ of P-SWV, S_5%-_SWV, and S_5%_-P-SWV in comparison and (b) Nyquist plots of P-SWV, S_5%_-SWV, and S_5%_-P-SWV in comparison. (c) CV curves of S_5%_-P-SWV at different scan rates; (d) Galvanostatic charge/discharge curves of S_5%_-P-SWV at different current densities.

In order to understand the contribution of lignin to the electrode capacitance, all conductive wood electrodes underwent identical electrochemical characterization using a three-electrode system with a 1 M NaCl electrolyte. In Figure 5a, the cyclic voltammetry (CV) curves of the P-SWV, S_5%_-SWV, and S_5%_-P-SWV electrodes are presented. Among these electrodes, the smallest CV curve area belonged to P-SWV, while the largest CV curve was observed for the S_5%_-P-SWV sample. This shows that P-SWV has the smallest charge storage capacitance, which is significantly increased in the S_5%_-SWV sample before reaching the highest values in the S_5%_-P-SWV electrode.^26,27^ The calculated capacitances for P-SWV, S_5%_-SWV, and S_5%_-P-SWV electrodes are 43 ± 3, 140 ± 10, and 315 ± 15 mF cm^–2^ at a scan rate of 5 mV s^–1^, respectively.^11,28^ Since S_5%_-SWV exhibited a capacitance three times higher than that of P-SWV, we can confirm that S-PEDOT has advantages over PEDOT/PSS in accessing and utilizing native lignin within the wood cell wall and in the central middle lamella. Moreover, a combination of these two polymers in S_5%_-P-SWV resulted in a capacitance that was seven times (P-SWV) and two times (S_5%_-SWV) higher than the capacitances of conductive wood prepared by using a single polymer. This suggests that synergy between these two polymers results in improved performance of conducting wood-based energy storage materials.

Importantly, all three electrodes in Figure 5a present a similar CV curve shape, showing a pair of oxidation and reduction reaction peaks located at potentials of about 0.6 and 0.4 V, respectively. These two peaks have previously been attributed to the redox reaction peaks of native lignin.^12,15^ These peaks exhibit the highest intensities in S_5%_-P-SWV, indicating that in this sample a large contribution to the total capacitance of the electrode comes from lignin that has been made accessible by the combined polymer treatment. This effective utilization is not observed in the samples treated only by either PEDOT/PSS or S-PEDOT since their corresponding CV curves show lower capacitance and less redox activity.

The electrochemical impedance (EIS) results presented in Figure 5b) have further indicated the better performance of the S_5%_-P-SWV electrode compared to S_5%_ -SWV and P-SWV samples. In general, Nyquist plots of all electrodes show mixed characteristics of both capacitive and redox-active materials, starting with a deviated arc at high frequencies before being elongated with a straight line at low frequencies.^12,28,29^ The highest impedances at both low and high frequency regions are displayed by S_5%_-SWV, suggesting poor ion diffusion and slow charge transport during the operation of this electrode.^30^ In contrast, the combination of S-PEDOT and PEDOT/PSS induced a conductive wood electrode (S_5%_-P-SWV) showing lower resistance and facilitating a better ion diffusion process. This enhancement is probably attributed to the high electronic-ionic conductivity of PEDOT/PSS and the electrical interconnection created by combining these two polymers in S_5%_-P-SWV. The important role played by PEDOT/PSS is further seen in the third sample, P-SWV, which has similar, although not quite as good, impedance characteristics as the sample that contains both PEDOT/PSS and S-PEDOT. By intercepting the Nyquist plots with the *x*-axis at high frequencies, the equivalent series resistance (ESR) of P-SWV, S_5%_-SWV, and S_5%_-P-SWV electrodes is obtained at values of 9.0, 12.8, and 5.8 Ω, respectively.^11,27,28^ The lowest resistance of S_5%_-P-SWV suggests that the interconnection design is a suitable approach for preparing a low EIS impedance electrode for electrochemical applications.

Since S_5%_-P-SWV has been shown to be the best electrode, its electrochemical properties were further examined to understand the electrode’s potential in energy storage applications. In Figure 5c, the CV curves of S_5%_-P-SWV present a similar curve shape at different scan rates, indicating the good reversibility of electrochemical processes, including the redox reactions.^12,31^ At a higher scan rate, the CV curve has a higher curve area, which is well aligned with the electrode’s charge/discharge behavior in Figure 5d, where discharge time is longer at a lower current density. The faster charge/discharge at a higher current density and the larger CV curve area at a higher scan rate also suggest that S_5%_-P-SWV is a promising electrode for energy storage devices with a good rate capability. Alongside its relatively low ESR, the capacitances of S_5%_-P-SWV electrodes were evaluated based on their CV curves’ area using eq S1 (Supporting Information),^28^ showing 315 ± 15, 264 ± 12, 222 ± 10, 197 ± 8, and 163 ± 8 mF cm^–2^ at scan rates of 5, 10, 20, 30, and 50 mV s^–1^, respectively. While the aim of our work has been to develop a multimaterial material approach to utilize the charge-storage capacity of lignin in conducting wood, the obtained capacitances are comparable to those of various conducting polymer/wood electrodes, despite differences in electrode characteristics, evaluation methods, and reporting metrics (see Table S1 and its discussion in the Supporting Information). We have found that using this approach, native lignin accounts for over 70% of the overall capacitance of the conductive wood electrode (Figure S6, Supporting Information), highlighting its significant contribution to charge storage in wood, a finding that has not been previously reported.

It is also worth noting that the charge/discharge profile of S_5%_-P-SWV resembles that of a battery-like electrode, as represented in Figure S7 (Supporting Information). For future reference, we used eq S2 (Supporting Information) to evaluate the specific capacity of S_5%_-P-SWV based on the total mass of active materials (∼1 mg of the PEDOT (excluding PSS) and S-PEDOT polymers and ∼4 mg of lignin in 1 cm^2^ electrode),^12^ resulting in a specific capacity of ∼19 mA h g^–1^ at a current density of ∼0.2 A g^–1^. The mass of polymers was determined by measuring the difference in mass between SWV and S_5%_-P-SWV samples, while the lignin content was determined using the Klason lignin method, as reported in previous studies.^12,21^

### 2.4 Effect of the Concentration of Monomer Solution

Although 5 wt % has been identified as the optimal monomer concentration^23^ for high-performance S-PEDOT preparation, this study also investigated the impact of a 1 wt % concentration on the resulting S-PEDOT-containing conductive woods. The electrochemical performance of P-SWV, along with its corresponding conductive woods prepared using 1 wt % (S_1%_-P-SWV) and 5 wt % (S_5%_-P-SWV) S-EDOT solutions, is presented in Figure S8a. In the figure, the area of the CV curves suggested that conductive wood prepared in a higher concentration of the S-EDOT monomer (5 wt %) has a higher capacitance than that prepared in a lower concentration (1 wt %). Indeed, the S_1%_-P-SWV conductive wood electrode presented a capacitance of 95 ± 5 mF cm^–2^ at a scan rate of 5 mV s^–1^, which is about three times lower than the capacitance of the S_5%_-P-SWV electrode. The lower capacitance is not only attributed to the possible lower polymer content in wood but also resulted from the poorer electrical performance of S-PEDOT prepared from a lower monomer content solution (1 wt %).^23^ Consequently, S_5%_-P-SWV, which presented the highest capacitance, was selected as the targeted conductive wood product in this work.

### 2.5 Effect of the Order in which the Two Polymers are Added

Figure 1 illustrates our method of preparing conductive wood, wherein PEDOT/PSS is introduced prior to the polymerization of S-PEDOT within the wood structure. This sequence of addition was found to be more effective than the reverse order (Figure S2b, Supporting Information), where SWV was first modified with S-PEDOT via in situ polymerization in a 5 wt % monomer solution before being coated with PEDOT/PSS. This version of conductive wood is denoted P-S_5%_-SWV, and its electrochemical performance is presented in comparison with the S_5%_-P-SWV electrode in Figure S8b. P-S_5%_-SWV exhibited a smaller CV curve area than S_5%_-P-SWV and thus has a poorer capacitance (156 ± 7 mF cm^–2^ at a scan rate of 5 mV s^–1^) compared to the capacitance of S_5%_-P-SWV (315 ± 15 mF cm^–2^ at the same scan rate). The capacitance of P-S_5%_-SWV is approximately the same as the capacitance of S_5%_-SWV, which reflects our observation that only a small amount of PEDOT/PSS was introduced in the impregnation step (the electrode mass is nearly unchanged). This observation indicates that reversing the sequence of polymer addition presented in Figure 1 is less suitable for the preparation of a high-capacitance electrode.

The cyclic stability of conductive wood electrodes was also examined and is presented in Figure S9, Supporting Information. While the capacitance retention of these electrodes is not ideal for energy storage applications (reaching up to 61% after 500 cycles), they demonstrate an improvement compared to reported values for similar systems, such as PEDOT/PSS/sulfonated wood (50% after 400 cycles)^12^ and polypyrrole/sulfonated wood (42% after 300 cycles).^21^ Given that native lignin accounts for over 70% of the total charge storage capacitance and that PEDOT polymers are electrochemically stable, the capacitance loss observed after 500 cycles in S_5%_-P-SWV can be attributed to the degradation of native lignin, which is a natural component of wood. The increase in capacitance retention and, more significantly, the more efficient utilization of native lignin capacity underscores the potential of our approach for the development of sustainable and enhanced-performance conductive wood electrodes.

While our primary focus is on developing an effective method to utilize native lignin, it is also worthwhile to consider the scalability of conductive wood and the associated challenges. The preparation of conductive wood involves several steps and requires various materials. Therefore, optimizing its performance necessitates careful selection of materials, such as the type of wood and conducting polymers, and the identification of optimal processing parameters,^12^ such as reaction time,^12^ temperature, and reactant concentrations,^21^ for wood pretreatment and in situ polymerization. We have selected several parameters for optimization, but a full optimization across the whole parameter space remains to be done. Despite these challenges, we believe that the scalability of conductive wood is feasible. WV is widely available in large quantities, and although PEDOT polymers are currently costly, they have already been synthesized and commercialized on a kilogram scale.^23^ Additionally, the conductive wood, specifically S_5%_-P-SWV, demonstrates excellent long-term stability. After more than a year of storage under ambient conditions, its performance remains consistent with that of freshly prepared conductive wood (Figure S6 and discussion in the Supporting Information).

## 3 Conclusions

In this study, we successfully introduced a systematic method for enhancing charge storage capacitance in conductive wood by incorporating polymer-based electrical interconnections into the wood structure to utilize lignin’s redox properties. This approach involved modification of the wood structure with PEDOT/PSS, followed by the introduction of S-PEDOT into the wood nanostructure in the wood cell wall and central middle lamella. The creation of electrical interconnections between PEDOT/PSS, lignin, and S-PEDOT was proven by visualizing their distribution through EDX and confocal Raman spectroscopy. This polymer-based interconnection not only included wood with electrical conductivity but also utilized native lignin capacity to the total charge storage capacitance. Accordingly, the obtained electrode showed a specific capacitance of 315 mF cm^–2^, which is contributed by both the polymers and native lignin. Notably, native lignin contributed over 70% of the total charge storage capacitance in the conductive wood, achieved with just a small fraction (6 wt %) of added PEDOT polymers. The obtained capacitance is also significantly higher than the capacitance of conductive wood modified only by either PEDOT/PSS (43 mF cm^–2^) or S-PEDOT (140 mF cm^–2^). The enhanced capacitance indicates the potential of this approach for designing and developing cost-effective and sustainable conductive wood, with the utilization of native lignin’s capacity, for electronic and energy storage applications.

## 4 Experimental Section

### 4.1 Materials

Natural pine veneers were sourced from Glimakra of Sweden AB. PEDOT/PSS (CLEVIOS PH 1000) was purchased from Heraeus Deutschland GmbH & Co. KG, Germany. (2,3-Dihydrothieno[3,4-*b*][1,4]dioxin-2-yl)methanol (HMEDOT, Sigma-Aldrich), 2,4-buthane sultone (Fujifilm Wako Pure Chemical), sodium hydride (NaH 60%, Sigma-Aldrich), sodium sulfite anhydrous (Na_2_SO_3_, Fisher Scientific), sulfuric acid (H_2_SO_4_, >95%, Fisher Scientific), Iron(II) sulfate heptahydrate (FeSO_4_·7H_2_O, Sigma-Aldrich), acetone (≥99.5%, VWR), dimethyl sulfoxide (DMSO, Sigma-Aldrich), tetrahydrofuran (anhydrous THF, Sigma-Aldrich), and ethanol (Sigma-Aldrich) were all used as received. Carbon felt was obtained from SGL Carbon, while carbon fibers, paraffin wax, and carbon paste were supplied by Sigma-Aldrich and used without further modification.

### 4.2 Synthesis of the S-EDOT Monomer

The synthetic procedure of the S-EDOT (sodium 4-[(2,3-dihydrothieno[3,4-*b*][1,4]dioxin-2-yl)methoxy]butane-2-sulfonate) monomer was adapted from the previously published protocol,^23^ in which S-EDOT was obtained via the functionalization of HMEDOT using 2,4-butane sultone. In this work, 394 mg of HMEDOT was first diluted in 2 mL of THF before dropwise adding the obtained solution into a reactor containing 72 mg of NaH (prepared from 120 mg of NaH, 60%). The reactor was placed in an ice bath while adding the HMEDOT solution. Subsequently, 2 mL of THF was added into the reactor, and the reaction solution was stirred in the ice bath for 15 min before undergoing a reflux reaction for 1 h. After that, a solution of 268 μL of buthane sultone diluted in 2 mL THF was dropwise added into the reactor, and the reflux reaction was continued overnight (16 h). The obtained reaction solution underwent quenching with methanol and several steps of precipitating, filtering, and drying before the final yellowish powder of the S-EDOT monomer was obtained. The molecular structure of synthesized S-EDOT was verified using ^1^H nuclear magnetic resonance (NMR). A synthetic diagram of S-EDOT is presented in Figure S1, and the NMR spectrum of the obtained S-EDOT monomer is presented in Figure S3.

### 4.3 WV Sulfonation

WVs were prepared by cutting them into dimensions of 3.0 × 1.0 × 0.075 cm^3^ (longitudinal × tangential × radial) and subjecting them to a vacuum while immersed in deionized water before undergoing chemical modification. Subsequently, the wet WVs (7 pine veneers of roughly 700 mg of dry weight) were sealed in an acid digestion vessel containing 16 mL of Na_2_SO_3_ solution (0.7 M) at pH 7, which was adjusted by adding H_2_SO_4_ at room temperature. The vessel was then transferred to an oven preheated to 80 °C and maintained at this temperature for 1 h. Following the impregnation step, the oven’s temperature was raised to 165 °C, and the reaction continued for 3 h. The resulting SWV product was designated as SWV. At the end of the reaction, the vessel was swiftly cooled in an ice water bath, and the modified SWV underwent thorough washing, first with acetone (3 times) and then with deionized water (3 times), all under vacuum conditions. SWV was then cut into three samples having a size of length × width = 1 cm × 1 cm for conductive wood preparation.

### 4.4 Conductive WV Preparation

#### 4.4.1 P-SWV (PEDOT/PSS-Modified SWV)

The process involved immersing SWV in a mixture of PEDOT/PSS and DMSO, followed by a drying step. To prepare the suspension, 6.0 g of DMSO was mixed with 100 g of PH1000, resulting in a mass ratio of roughly 1:5 for PEDOT/PSS and DMSO. For the preparation of P-SWV, 6 SWV samples (length × width = 1 cm × 1 cm) were fully soaked in a 25 mL Petri dish containing 16.0 g of the suspension. This Petri dish was vacuumed in a desiccator for 15 min before transferring into an air-drying oven set at 75 °C for 10 h until the samples were completely dry. The final conductive wood (P-SWV) products were obtained by completely removing the polymer layers that had aggregated on the surface of the dried samples (Figure S2, Supporting Information).

### 4.5 In Situ Polymerization of SEDOT in WV

#### 4.5.1 S_5%_-SWV

Each air-dried SWV wood sample (size of length × width = 1 cm × 1 cm) was impregnated in the S-EDOT solution prepared by diluting 5 mg (15.1 μmol) of the S-EDOT monomer and 5 mg (18 μmol) of FeSO_4_·7H_2_O in 85 μL of H_2_SO_4_ 1 M. The use of 5 mg S-EDOT is to obtain a 5 wt % monomer solution, which is composed of the S-EDOT solution and 12.5 μL of (NH_4_)_2_SO_3_ (42 wt %) solution, prior to the polymerization. Subsequently, the wood sample was left to absorb the monomer solution for 10 min until the wood was fully absorbed with the solution. 12.5 μL of (NH_4_)_2_SO_3_ (42 wt %) solution was then distributed all over the wood sample, allowing it to spread and penetrate throughout the wood structure to induce the polymerization of S-EDOT. The reaction was left overnight in an ambient atmosphere. The obtained S-PEDOT-modified SWV (S_5%_-SWV) was then washed in deionized water until the washed solution reached a neutral pH. The sample was then vacuum-dried to obtain the final conductive wood.

The timeline for the in situ polymerization process is as follows: (i) the wood is dried for 20 h; (ii) the S-EDOT monomer is dissolved by stirring for 15 min to form the solution; (iii) the dried wood is impregnated in the S-EDOT solution for 10 min to ensure full absorption; (iv) the in situ polymerization is performed and maintained for 15 h; (v) the resulting conductive wood is washed and vacuum-dried for 15 h to obtain the final product.

#### 4.5.2 S_5%_-P-SWV

The in situ polymerization of S-EDOT (a 5 wt % monomer solution) in P-SWV wood samples (length × width = 1 cm × 1 cm) was carried out using the same protocol as used for the preparation of S_5%_-SWV. The obtained conductive wood is S_5%_-P-SWV, which is the optimal conductive wood product in this work.

#### 4.5.3 S_1%_-P-SWV

Since 5 wt % was reported as an optimal S-EDOT concentration^23^ for preparing a high-conductive S-PEDOT polymer, a 5 wt % S-EDOT solution was employed to prepare the final conductive woods in this work. However, to understand the effect of monomer concentration on the conductive wood performance, a lower concentrated S-EDOT monomer solution (1 wt %) was used to prepare S_1%_-P-SWV using the same protocol used for the preparation of S_5%_-P-SWV. Instead of using 5 mg of S-EDOT (5 wt %), 1 mg of S-EDOT was used to prepare a 1 wt % monomer solution in this preparation.

#### 4.5.4 P-S_5%_-SWV

To examine the effect of the order in which PEDOT/PSS and S-PEDOT are added, P-S_5%_-SWV was prepared using reversed polymers, adding order to the preparation of S_5%_-P-SWV. Particularly, S_5%_-SWV was first prepared as described above before being added with PEDOT/PSS following the same protocol as the addition of PEDOT/PSS into SWV (P-SWV). Further information is presented in Figure S2, Supporting Information.

### 4.6 Characterization Methods

The characterization methods are presented in detail in the Supporting Information.

## Data Availability

Data are available from the authors upon reasonable request.

## Supporting Information Available

Synthetic procedures for S-EDOT and conductive wood using S-PEDOT; NMR spectrum of S-EDOT; EDX compositional analysis of different PEDOT-based conductive wood samples; confocal Raman microscopy of S_5%_-P-SWV conductive wood; CV curves of S-PEDOT film and S_5%_-P-SWV; discussion of S_5%_-P-SWV stability over time; discussion of S_5%_-P-SWV charge storage performance in comparison with previously reported conductive wood and a table with the data for said discussion; charge/discharge curves of S_5%_-P-SWV; and CV curves and cyclic stability of different S-PEDOT-based conductive wood samples (PDF)
