# Supplementary material for: Interconnecting EDOT-Based Polymers with Native Lignin toward Enhanced Charge Storage in Conductive Wood
Source: ACS Appl Mater Interfaces. 2024 Dec 3;16(49):68416–25. doi: 10.1021/acsami.4c16298 (PMC11647749; doi:10.1021/acsami.4c16298)
Supplement: Supplementary file 1 — am4c16298_si_001.pdf [file am4c16298_si_001.pdf]

Supporting Information

Interconnecting EDOT-based Polymers with Native Lignin towards Enhanced Charge-Storage in Conductive Wood

Van Chinh Tran* [1,2,4], Gabriella Mastantuoni [5,6], Jonas Garemark[7], Christopher H. Dreimol[7,8], Xin Wang[9,10], Magnus Berggren[1,2,3], Qi Zhou [5,6], Renee Kroon* [1,2], Isak Engquist* [1,2].

[1] *Laboratory of Organic Electronics, Department of Science and Technology, Linköping University, SE-601 74 Norrköping, Sweden.*

[2] *Wallenberg Wood Science Center, Department of Science and Technology, Linköping University, SE-601 74 Norrköping, Sweden.*

[3] *Wallenberg Initiative Material Science for Sustainability, Department of Science and Technology, Linköping University, SE-601 74 Norrköping, Sweden.*

[4] *Department of Chemistry, Massachusetts Institute of Technology, 77 Massachusetts Avenue, Cambridge, Massachusetts 02139, United States.*

[5] *Division of Glycoscience, Department of Chemistry, KTH Royal Institute of Technology, AlbaNova University Centre, 106 91 Stockholm, Sweden.*

[6] *Wallenberg Wood Science Center, Department of Fiber and Polymer Technology, KTH Royal Institute of Technology, 100 44 Stockholm, Sweden.*

[7] *Wood Materials Science, Institute for Building Materials, ETH Zürich, 8093, Zürich, Switzerland.*

[8] *Cellulose & Wood Materials Laboratory, Empa, 8600, Dübendorf, Switzerland.*

[9] *Division Digital Systems, Department Smart Hardware, Unit Bio- and Organic Electronics, RISE Research Institutes of Sweden, 602 33 Norrköping, Sweden.*

[10] *Digital Cellulose Center, RISE, 602 33 Norrköping, Sweden.*

Corresponding authors: Prof. Isak Engquist (isak.engquist@liu.se), Prof. Renee Kroon (renee.kroon@liu.se), Dr. Van Chinh Tran (tran.van.chinh@liu.se).

1. Experimental

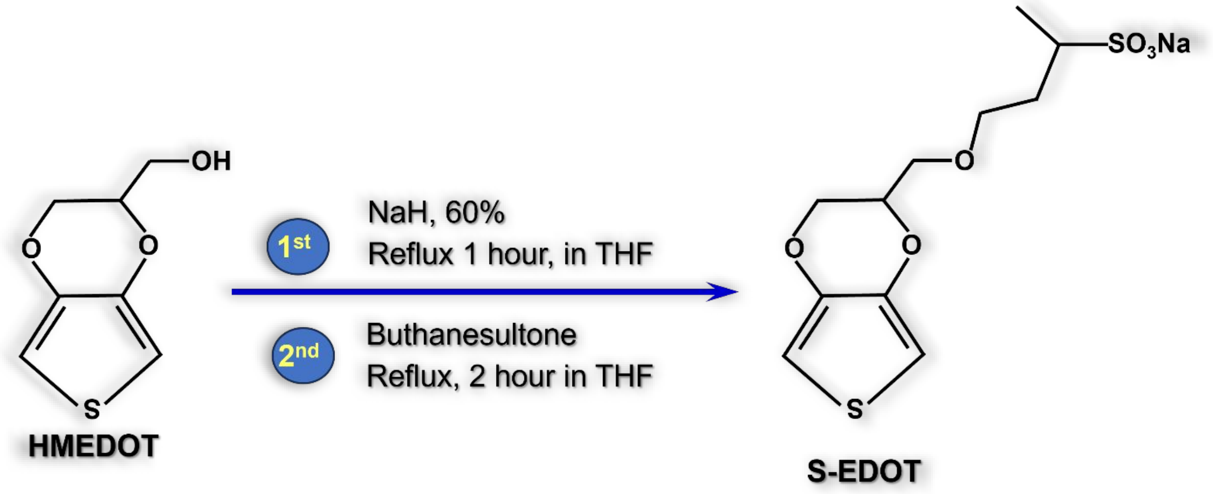

Figure S1: Synthetic route of S-EDOT [1].

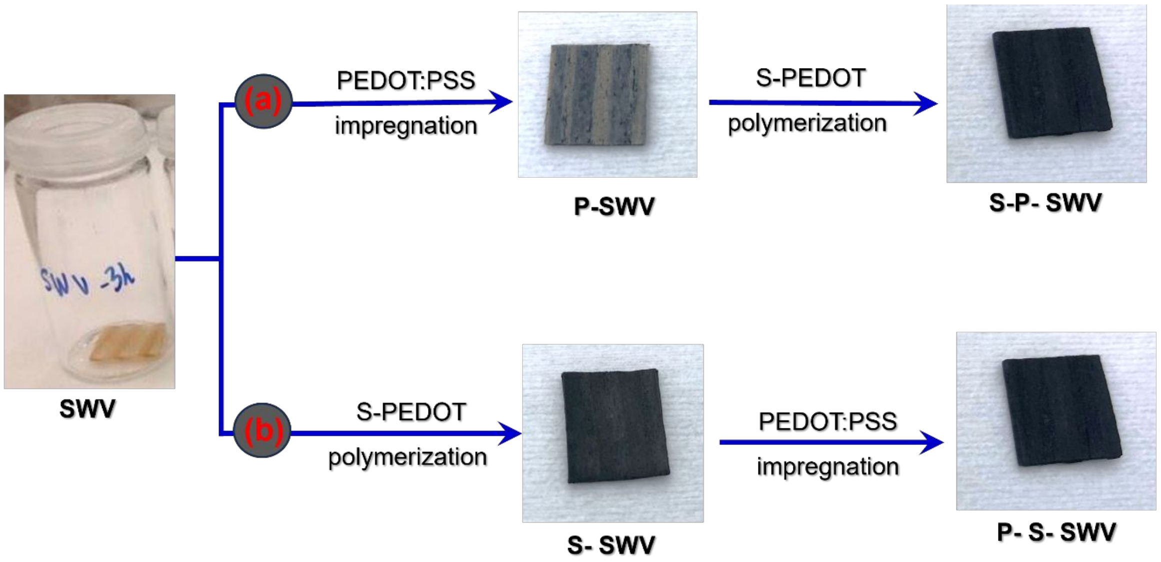

Figure S2: Schematic illustration of conductive woods preparation: a) Depiction of the method described in Figure 1, employed for the creation of targeted conductive woods such as **S-P-SWV**. b) Modified approach for P-S-SWV preparation, showcasing a reversal in the sequence of adding PEDOT:PSS and S-PEDOT compared to the procedure outlined in Figure 1.

2. Characterizations

Chemical, structural, and morphological analysis:

The chemical structure of S-EDOT monomer was characterized using the Benchtop NMR (Model: Spinsolve Carbon 80 MHz from Magritek). The monomer was diluted in deuterium oxide (D_2O) before the measurement.

The microstructure of the wood samples was examined using field emission scanning electron microscopy (FESEM) with a Hitachi S-4800 instrument from Japan, operating at an acceleration voltage of 1 kV and a working distance of 8 mm. To enhance conductivity, the samples were coated with a platinum/palladium layer using a Cressington 208HR sputter coater from the UK. Energy-dispersive X-ray spectroscopy (EDX) was conducted using an Oxford Instruments X-MAX N 80 system from the UK, operating at an acceleration voltage of 6 kV and a working distance of 15 mm. Prior to analysis, clean sections of the sample's surface were obtained using a sliding microtome (SM 2010R) from Leica, Germany.

Confocal Raman spectroscopy measurement of wood samples following the same previously published protocol[2]. Typically, Raman spectroscopy was performed using a confocal Raman microscope (Renishaw InVia) equipped with a 532 nm laser and an 1800 l/mm-1 grating. An oil immersion objective (100x, NA 1.3) with microscope immersion oil (Roth, ρ=1.03) was utilized. Samples were prepared by microtoming 180 μm cross-section slices of the wood veneer used in the conductive wood preparation (paper IV). These slices were placed on a glass slide with deuterium oxide, covered with a glass coverslip (0.16 mm thickness), and sealed with nail polish. Mapping parameters were unique to each individual sample, in general, the power ranged between 1.5 to 3.5 mW while the exposure time ranged from 0.8 and 2s for 3 to 7 accumulations over a spectral range of 391-2112 cm^{-1}. A total area of 15 x 15 μm^2 with a step width of 200 nm (Map image acquisition mode) was mapped for each sample while the acquired data was baseline corrected afterward and filtered from cosmic ray signal using the software Wire 4.4 (Renishaw, UK). Chemical analysis of the spectroscopic data was performed using Cytospec (v. 2.00.07), integrated into MatLab. Univariate color-represented chemical images were achieved by integrating over specific intensities for PEDOT: PSS (1430–1450 cm^{-1}), and lignin (1584–1618 cm^{-1}).

Electrical and Electrochemical measurement:

The conductive wood electrodes were prepared for electrochemical measurements following the same procedure as in our prior works[3, 4]. Typically, the 1 cm^2 conductive wood was connected to carbon fibers using carbon paste, followed by wrapping a section of the carbon fiber with paraffin wax and Kapton tape. Electrochemical measurements were conducted in a three-electrode system configuration utilizing a potentiostat/galvanostat (BioLogic, SP-200) connected to a computer. Sample capacitance (Farad, F) was determined using the **equation S1** [5, 6]: $C = \frac{1}{v\Delta V}\int_{V1}^{V2} idV = \frac{A_i}{2 \times v \times \Delta V}$, where i represents the charge/discharge current (A), A_i is the integral area of the CV curve, v is the scan rate (mV s^{-1}), and ΔV is the working potential of the discharge process.

The electrode capacity can also be calculated using the **equation S2** [5]: $C_s = Q/m\Delta V$, where Q is the total charge measured for the discharge process (C), m is the total mass of active materials (PEDOT and S-PEDOT) (g), and ΔV is the working potential of the discharge process.

At least three samples were prepared for each type of conductive wood electrode for electrical and electrochemical measurements.

3. Results and Discussion

***NMR result*:** The obtained monomer displayed a 1H NMR spectrum (Figure S3) with nearly identical peaks to those reported in a previously published study on the synthesis of S-EDOT [1], confirming the successful synthesis of the S-EDOT monomer in this research.

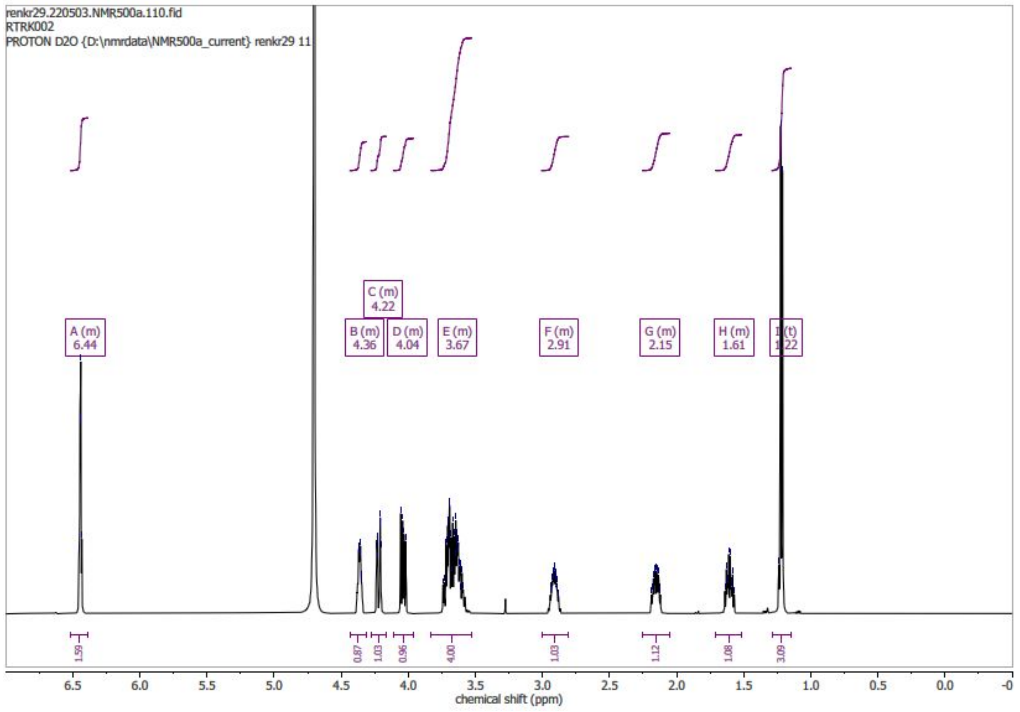

Figure S3: NMR spectrum of synthesized S-EDOT monomer.

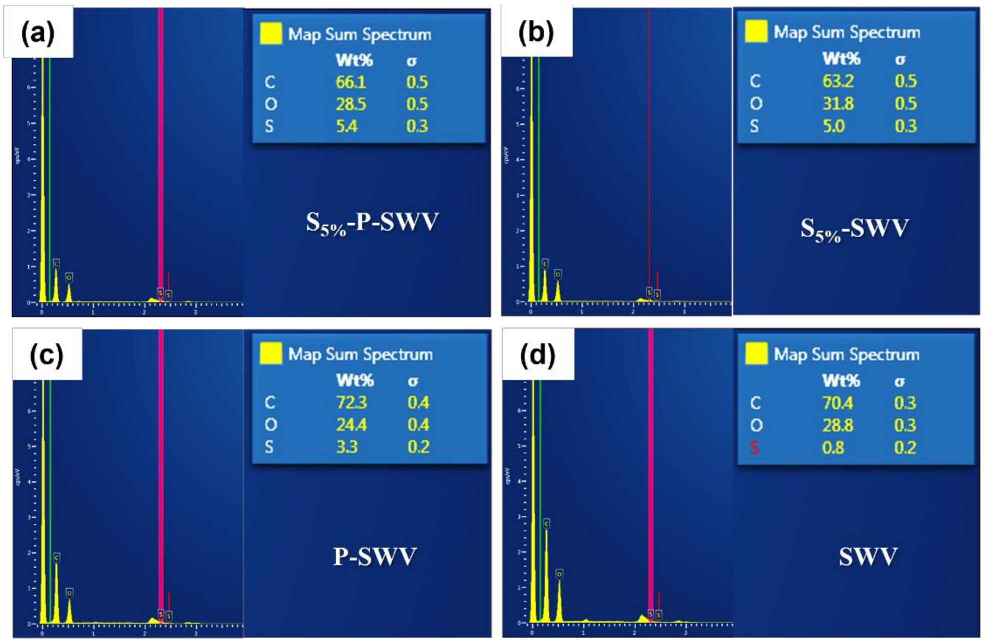

Figure S4: EDX measurement results: Atomic percentages of oxygen (O) carbon (C), and sulfur (S) in a) $S_{5\%}$-P-SWV, b) $S_{5\%}$-SWV, c) P-SWV, d) SWV.

***Confocal Raman microscopy*:**

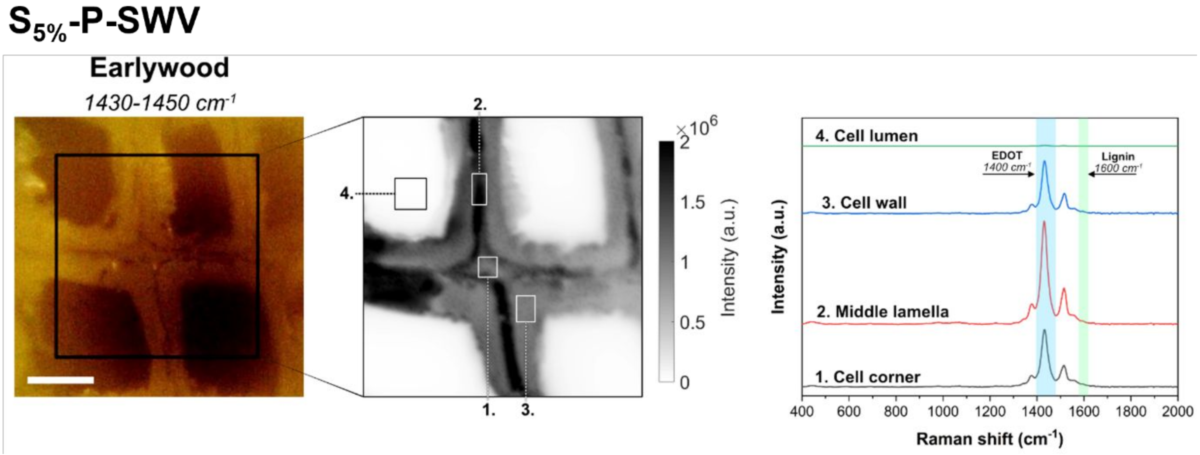

Figure S5: Confocal Raman microscopy of earlywood $S_{5\%}$-P-SWV sample showing a microscope image accompanied by a Raman map showing the EDOT signal in black followed by signals from the individual regions of interest which were integrated from the areas of the map.

Stability of conductive wood electrodes over time:

To assess the long-term stability of conductive wood electrodes, we measured $S_{5\%}$-P-SWV samples that were prepared and stored under ambient conditions for one year. These samples maintained stable electrical conductivity and electrochemical performance, comparable to the results of freshly prepared electrodes. This stability underscores the durability of the electrodes over time. Specifically, the one-year-old $S_{5\%}$-P-SWV samples retained a conductivity of approximately 145 S/m and a charge storage capacitance of ~330 mF/cm^2, as shown by the CV curves in Figure S6a.

We also explored the contribution of native lignin to the overall charge storage capacity of the electrodes. PEDOT:PSS [7] and S-PEDOT (Figure S6b) are recognized as capacitive materials, and lignin undergoes redox reactions within the potential range of 0.3 to 0.7 V. Therefore, we measured the CV of $S_{5\%}$-P-SWV within a reduced potential range of -0.2 to 0.1 V (Figure S6a). The resulting CV curve displayed a nearly rectangular shape, indicative of the capacitive behavior of PEDOT:PSS and S-PEDOT. The capacitance, calculated using Equation S1, was found to be 91 mF/cm^2, accounting for approximately 28% of the total charge storage capacity of the conductive wood. This finding suggests that native lignin contributes over 70% of the total capacitance, emphasizing its crucial role in enhancing energy storage efficiency in wood-based electrodes.

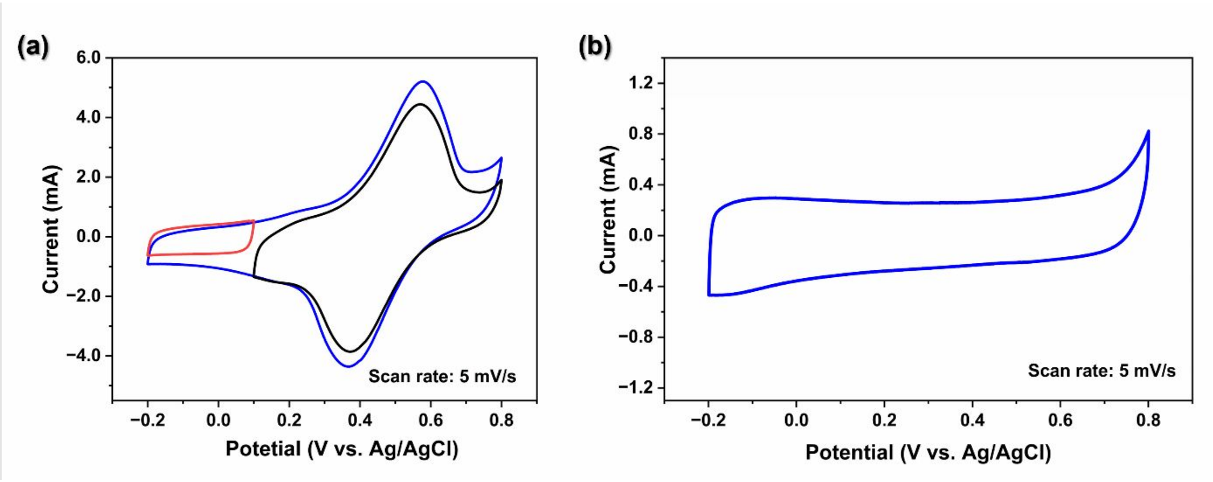

Figure S6. a) CV curves of the 1 year old $S_{5\%}$-P-SWV electrode measured over different potential window; **b)** The CV curve of an S-PEDOT film (1 mg/cm^2) prepared from the 1 wt% S-PEDOT suspension.

<u>Charge storage performance of $S_{5\%}$-P-SWV</u>**<u>:</u>**

Before comparing the performance of our conductive wood ($S_{5\%}$-P-SWV) with previously reported conductive woods, it's important to recognize that these comparisons can be inconsistent due to differences in how energy storage data are reported and interpreted[5]. This issue arises particularly with the reported charge storage data of conductive wood electrodes for energy storage devices. As summarized in Table S1, capacitance is calculated using different methods and reported in various formats in each study[8-12]. Researchers have measured capacitance from CV[5, 8-11], or charge/discharge profiles[5, 8-12] by integrating the area under the curves or calculating based on discharge time. While the integration method is widely accepted for all types of electrodes, calculating based on discharge time is only suitable for EDLC supercapacitor electrodes[5]. However, this distinction is often overlooked, leading to inappropriate comparisons among electrodes. Moreover, when researchers report areal- or mass-specific capacitance, they often ignore the electrodes' thicknesses or the mass of native lignin (a wood redox component)[8-12]. This oversight results in incorrect capacitance calculations and comparisons. Another point of contention is whether charge storage data should be reported as capacitance (F g^{-1}) or capacity (mAh g^{-1}) when studying redox-active materials for supercapacitors and battery electrodes. The latter is increasingly gaining acceptance[5].

Table S1: Energy Storage Data for Noncarbonized Conductive Woods in the Literature:

**Conductive wood electrodes**	**Active materials**	**Reported data**	**Capacitance - Calculation formulas**	**Native lignin contribution determined**	**Ref.**
PANI /RGO/wood<br>**Or**<br>PPy /RGO/wood	- LS<br>- PANI **or** PPy	~0.8 F cm^{-2} at 50 mV s^{-1}, 932 F g^{-1} at 2.5 mA cm^{-2}<br>**Or**<br>~0.4 F cm^{-2} at 50 mV s^{-1}, 848 F g^{-1} at 2.5 mA cm^{-2}	$C = \frac{\int IdV}{vA\Delta V}$ (F cm^{-2})<br>$C = \frac{I\Delta t}{m\Delta V}$ (F g^{-1})	No	S[8]
MWCNT/PANI/ Wood	- MWCNT<br>- PANI	0.27 F cm^{-2} at 2 mV s^{-1}	$C = \frac{\int IdV}{2vA\Delta V}$ (F cm^{-2})	No	S[9]
PPy/wood aerogel	- PPy	7.68 F cm^{-2} at 1 mA cm^{-2}<br>**Or**<br>206 F g^{-1} at 1 mA cm^{-2}	$C=\frac{I\Delta t}{A(\Delta V\text{-}IR)}$ (F cm^{-2})<br>$C=\frac{I\Delta t}{m(\Delta V\text{-}IR)}$ (F g^{-1})	No	S[10]
PPy/wood	- PPy	0.61 F cm^{-2} at 1 mV s^{-1}<br>**Or**<br>408 F g^{-1} at 0.1 mA cm^{-2}	$C=4\frac{\int IdV}{vA\Delta V}$ (F cm^{-2})<br>$C=4\frac{I\Delta t}{m\Delta V}$ (F g^{-1})	No	S[11]
LS/PPy/wood Symmetric supercapcitor device	- LS<br>- PPy	1.06 F cm^{-2} at 1.0 mA cm^{-2}	$C=2\frac{I\Delta t}{A\Delta V}$ (F cm^{-2})	No	S[12]
S-PEDOT/ PEDOT:PSS/wood	- Native lignin<br>- PEDOT:PSS<br>- S-PEDOT	0.315 F cm^{-2} at 5 mV s^{-1}<br>**Or**<br>~19 mAh g^{-1} at ~0.2 A g^{-1}	$C=\frac{1}{Av\Delta V}\int_{V1}^{V2} idV = \frac{A_i}{2 \times v \times \Delta V}$ (F cm^{-2})<br>Or<br>$C=\frac{Q}{m(\Delta V)}$ (mAh g^{-1})	Yes 70%	Our work

Abbreviations:
LS: Lignosulfonate; **PANI**: polyaniline; **RGO**: reduced graphene oxide; **MWCNT**: multiple-walled carbonanotubes.
$\boldsymbol{A}$: area of electrode (cm^2); $\boldsymbol{m}$: mass of active materials (g); $\boldsymbol{I}$ **or** $\boldsymbol{i}$: Current density (A/cm^2 or A/g), $\boldsymbol{v}$: scan rate (V/s); $\boldsymbol{A_i}$: integrated area under the CV curve; $\boldsymbol{\Delta t}$:discharge time (s) ; $\boldsymbol{\Delta V}$: Working potential window (V); ***IR***: potential drop (V).

In 2019, Gogotsi Y. and colleagues proposed guidelines for reporting energy storage data, which have been widely accepted in the research community[5]. We followed their recommended

calculation methods and reporting standards for our capacitance and capacity data. Although making direct comparisons with previously reported conductive electrodes can be challenging, our conductive wood electrode's performance is comparable to other reported works. Using the same calculation methods, our $S_{5\%}$-P-SWV electrode shows a higher capacitance of 0.315 F cm^{-2} at a scan rate of 5 mV s^{-1} compared to MWCNT/PANI/Wood (0.27 F cm^{-2} at 2 mV s^{-1})[9] and PEDOT:PSS/wood (0.04 F cm^{-2} at 5 mV s^{-1})[3]. When recalculating our data using different methods from other studies, we find that our electrode's capacitance usually remains similar. For example, using the equations from ref. S11 our electrodes achieve a capacitance of 1.26 F cm^{-2} at 5 mV s^{-1}, outperforming the PPy/wood electrode's 0.61 F cm^{-2} at 1 mV s^{-1} [11]. However, when using different calculation methods, some reported electrodes show higher or comparable capacitance to ours. Using the equations from refs. S8[8], S10[10], and S12[12], our electrode's capacitances are 0.63 F cm^{-2} at 5 mV s^{-1}, ~71 F g^{-1} at 1 mA cm^{-2}, and 0.7 F cm^{-2} at 1 mA cm^{-2}, respectively. These values are lower or comparable to the capacitances reported in SI8[8], SI10[10], and S12[12] in Table S1. While the mass of active materials is often reported in conductive wood research, the weight percentage of these materials relative to the total wood electrode mass, or volume, is rarely discussed. To better assess the efficiency of conductive wood preparation methods, we propose that capacitance normalized by the weight percentage of added materials can serve as a useful metric. To make the comparison as fair as possible, all the capacitance values have been scaled to represent a sample of 0.75 mm thickness.Using this way of benchmarking, we achieved a capacitance of 315 mF/cm^2 using approximately 6 wt% added polymers, yielding 52.5 mF/cm^2 per wt%. This slightly exceeds previous studies, which reported capacitances of 1710 mF/cm^2 with 35 wt% PPy (48.9 mF/cm^2 per wt%)[4] and 1747 mF/cm^2 with 41.4 wt% PANI (42.2 mF/cm^2 per wt%)[13].

Since our electrodes are made of both capacitive and redox-active materials, we also here wish to report the energy storage capacity in mAh g^{-1} for future reference. The $S_{5\%}$-P-SWV electrode has a charge storage capacity of approximately 19 mAh g^{-1} at a current density of 0.2 A g^{-1}.

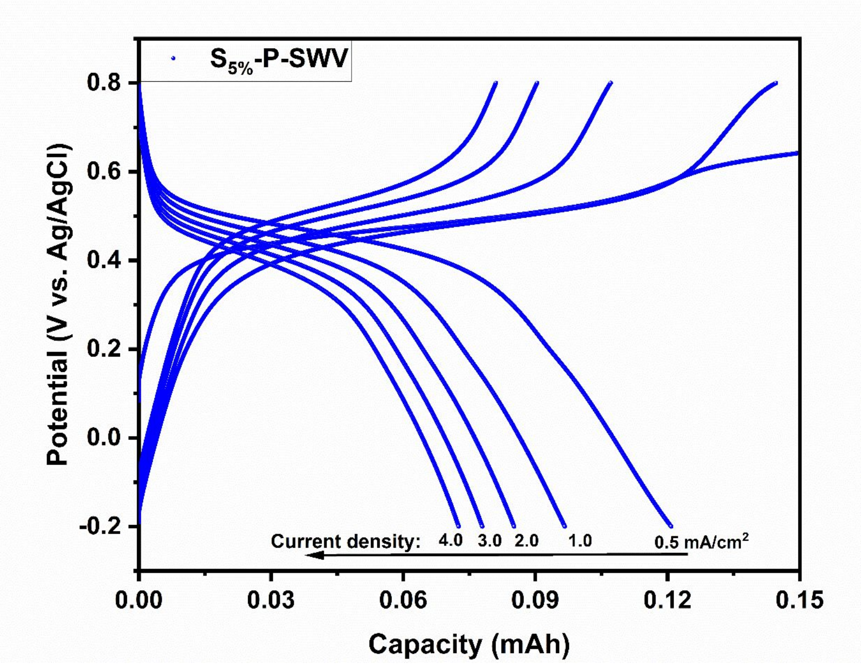

Figure S7: Charge/discharge curves of $S_{5\%}$-P-SWV at different current densities.

<u>Effect of monomer solution concentration and the adding order of the two polymers on the performance of conductive wood electrodes:</u>

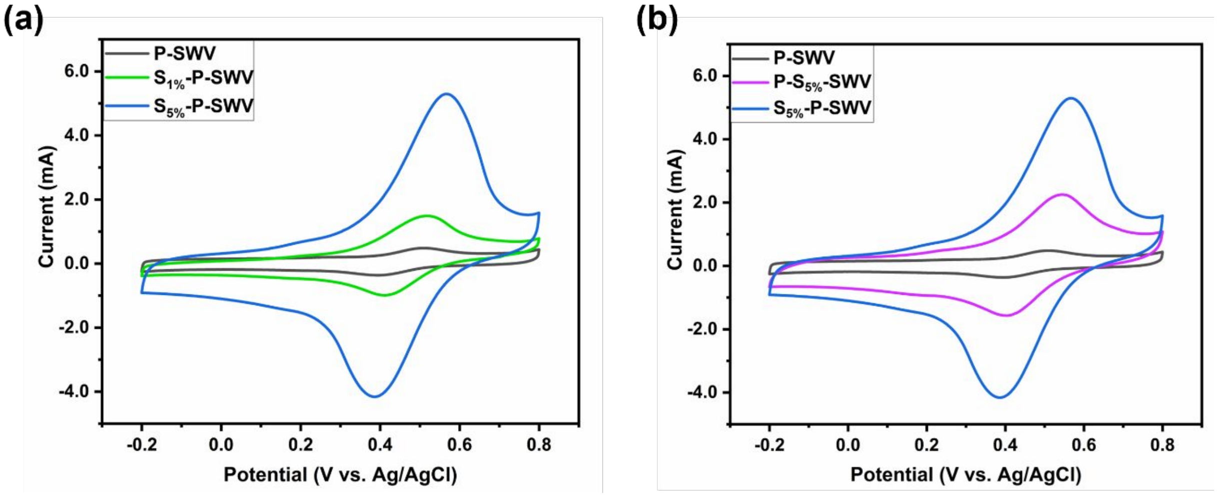

Figure S8. a) CV curves of P-SWV, $S_{1\%}$-P-SWV, and $S_{5\%}$-P-SWV electrodes at a scan rate of 5 mV s^{-1}; **b)** CV curves of P-SWV, P-$S_{5\%}$-SWV, and $S_{5\%}$-P-SWV electrodes at a scan rate of 5 mV s^{-1}.

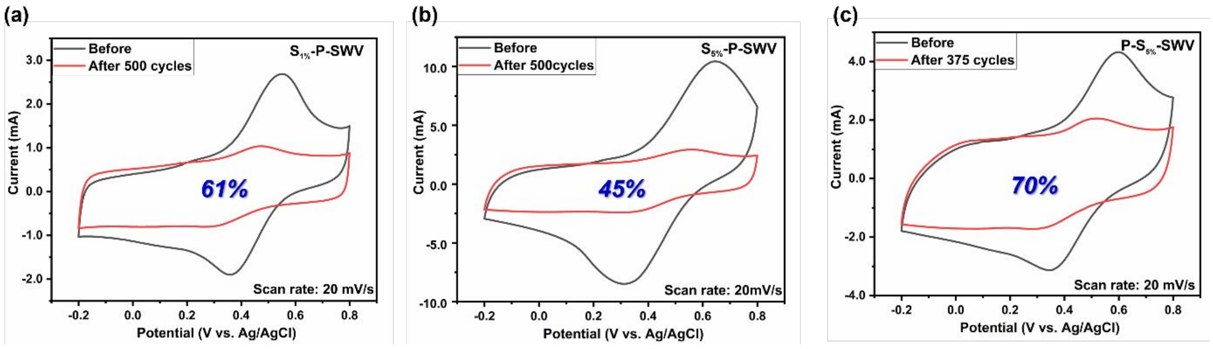

Figure S9. Capacitance retention of a) $S_{1\%}$-P-SWV, b) $S_{5\%}$-P-SWV, c) P-$S_{5\%}$-SWV electrodes measured using CV at a scan rate of 20 mV s^{-1}.

References:

(1) Yano, H.; Kudo, K.; Marumo, K.; Okuzaki, H. Fully soluble self-doped poly(3,4-ethylenedioxythiophene) with an electrical conductivity greater than 1000 S cm(-1). *Sci. Adv.* **2019**, *5* (4), eaav9492.

(2) Garemark, J.; Perea-Buceta, E.; Felhofer, M.; Chen, B.; Ruiz, M. F. C.; Sapouna, I.; Gierlinger, N.; Kilpelainen, I. A.; Berglund, L. A.; Li, Y. Y. Strong, Shape-Memory Lignocellulosic Aerogel via Wood Cell Wall Nanoscale Reassembly. *ACS Nano* **2023**, *17* (5), 4775-4789.

(3) Tran, V. C.; Mastantuoni, G. G.; Belaineh, D.; Aminzadeh, S.; Berglund, L. A.; Berggren, M.; Zhou, Q.; Engquist, I. Utilizing native lignin as redox-active material in conductive wood for electronic and energy storage applications. *J. Mater. Chem. A* **2022,** 10 (29), 15677-15688.

(4) Mastantuoni, G. G.; Tran, V.; Engquist, I.; Berglund, L. A.; Zhou, Q. In Situ Lignin Sulfonation for Highly Conductive Wood/Polypyrrole Porous Composites. *Adv. Mater. Interfaces* **2022,** (10), 2201597.

(5) Mathis, T. S.; Kurra, N.; Wang, X. H.; Pinto, D.; Simon, P.; Gogotsi, Y. Energy Storage Data Reporting in Perspective-Guidelines for Interpreting the Performance of Electrochemical Energy Storage Systems. *Adv. Energy Mater.* **2019**, *9* (39), 1902007.

(6) Tran, V. C.; Mastantuoni, G. G.; Zabihipour, M.; Li, L.; Berglund, L.; Berggren, M.; Zhou, Q.; Engquist, I. Electrical current modulation in wood electrochemical transistor. *PNAS* **2023**, *120* (18), e2218380120.

(7) Volkov, A. V.; Wijeratne, K.; Mitraka, E.; Ail, U.; Zhao, D.; Tybrandt, K.; Andreasen, J. W.; Berggren, M.; Crispin, X.; Zozoulenko, I. V. Understanding the Capacitance of

PEDOT:PSS. *Adv Funct Mater* **2017**, *27* (28), 1700329. Tran, V. C.; Mastantuoni, G. G.; Belaineh, D.; Aminzadeh, S.; Berglund, L. A.; Berggren, M.; Zhou, Q.; Engquist, I. Utilizing native lignin as redox-active material in conductive wood for electronic and energy storage applications. *J. Mater. Chem. A* **2022**, 15677-15688.
(8) Lyu, S. Y.; Chen, Y. P.; Han, S. J.; Guo, L. M.; Yang, N.; Wang, S. Q. Natural sliced wood veneer as a universal porous lightweight substrate for supercapacitor electrode materials. *Rsc Adv.* **2017**, *7* (86), 54806-54812.
(9) Ke, S. Q.; Xie, D.; Zhang, K.; Cheng, F. C.; Wu, Y. Q. Ultraflexible all-in-one supercapacitors with high capacitance and ultrastable cycle performance enabled by wood cellulose network. *Mater. Adv.* **2022**, *3* (4), 2026-2036.
(10) He, W.; Qiang, H.; Liang, S.; Guo, F. Y.; Wang, R.; Cao, J. Z.; Guo, Z. H.; Pang, Q. Y.; Wei, B. R.; Sun, J. W. Hierarchically porous wood aerogel/polypyrrole (PPy) composite thick electrode for supercapacitor. *Chem. Eng. J.* **2022**, *446 (22), 137331.*
(11) Lv, S. Y.; Fu, F.; Wang, S. Q.; Huang, J. D.; Hu, L. Novel wood-based all-solid-state flexible supercapacitors fabricated with a natural porous wood slice and polypyrrole. *RSC Adv.* **2015**, *5* (4), 2813-2818.
(12) Zhang, Z. C.; Yu, C. Y.; Peng, Z. Y.; Zhong, W. B. Mechanically stiff and high-areal-performance integrated all-in-wood supercapacitors with electroactive biomass-based hydrogel. *Cellulose* **2021**, *28* (1), 389-404.
(13) Si, R. R.; Luo, H. G.; Pu, J. W. Construction of wood-PANI supercapacitor with high mass loading using "pore-making, active substance-filling, densification" strategy. *J. Colloid Interface Sci.* **2024**, *662*, 58-68.
